# Noncontiguous multifocal *Brucella* spondylodiscitis with paravertebral abscess: a case report

**DOI:** 10.1186/s13256-022-03544-y

**Published:** 2022-11-03

**Authors:** Sarah Gerges, Alessandro Khoury, Souheil Hallit, Fadi Hoyek, Rabih Hallit

**Affiliations:** 1 School of Medicine and Medical Sciences, Holy Spirit University of Kaslik, P.O. Box 446, Jounieh, Lebanon; 2 Faculty of Medicine, American University of Beirut, Beirut, Lebanon; 3 Psychology Department, College of Humanities, Effat University, Jeddah, 21478 Saudi Arabia; 4 Research Department, Psychiatric Hospital of the Cross, Jal Eddib, Lebanon; 5 Department of Orthopedics, Notre Dame des Secours University Hospital Center, Street 93, Byblos, Postal Code 3 Lebanon; 6 Department of Infectious Disease, Notre Dame des Secours University Hospital Center, Street 93, Byblos, Postal Code 3 Lebanon; 7 Department of Infectious Disease, Bellevue Medical Center, Mansourieh, Lebanon

**Keywords:** Brucellosis, Spondylodiscitis, Multifocal, Paravertebral abscess, Lebanon, Case report

## Abstract

**Background:**

Human brucellosis is the most frequently contracted zoonotic infection worldwide. Although being an old disease that carries minimal risks of mortality, it remains a source of considerable sequelae and disability. However, noncontiguous multifocal spinal involvement is an exceptional presentation of brucellosis; additionally, an associated paravertebral abscess is extremely rare.

**Case presentation:**

This paper focuses on a 67-year-old Lebanese woman with noncontiguous multifocal *Brucella* spondylodiscitis, involving the T12–L1 and L3–L4 segments, with paravertebral abscess formation. She presented with a 3-week history of acute severe lumbar back pain, radiating to the lower extremities and associated with impaired mobility and lower extremity weakness. The patient complained of night sweating but had no fever. No lymphadenopathy, hepatomegaly, or splenomegaly could be observed. She had painful percussion of the lumbar spine, painful passive mobilization, and paravertebral tenderness, yet her neurological examination was completely normal. BrucellaCapt test was positive at a titer of 1/5120 (reference range 1/180). The patient was treated with an inpatient regimen for 2 weeks, which was followed by an outpatient oral antibiotic regimen with doxycycline, rifampin, and ciprofloxacin to complete a total treatment duration of 3 months. Magnetic resonance imaging was performed at the end of the treatment and showed a complete resolution of the paravertebral abscess.

**Conclusion:**

Noncontiguous multifocal *Brucella* spondylodiscitis with paravertebral abscess is an extremely rare presentation. It may be effectively managed by antibiotic therapy, without surgery or drainage, in the absence of neurological complications. Nonetheless, the principal challenge to an efficient management is establishing the diagnosis of *Brucella* in the first place. In endemic countries, a strong suspicion of spinal involvement of brucellosis should be elicited in front of back pain presentations—even in the absence of fever and other related symptoms.

## Background

Human brucellosis is the most frequently contracted zoonotic infection worldwide, with an incidence exceeding 500,000 cases recorded annually until 2006. Although being an old disease that carries minimal risks of mortality, it remains a source of considerable sequelae and disability [1]. The involved pathogens are *Brucella* spp., Gram-negative coccobacilli. Acquisition usually occurs through consumption of contaminated milk/dairy products or direct contact with infected animals [2]. Despite the remarkable epidemiological decline in developed countries, brucellosis remains quite common in many countries, particularly in the Mediterranean region. Human brucellosis presents as an acute febrile disease; nevertheless, it might also be subclinical, and chronic infections with relapses might occur—even months succeeding an apparently successful treatment. This infection causes lesions in a wide range of organs and occasionally leads to complications that include—but are not limited to—arthritis, spondylitis, uveitis, meningitis, and epididymoorchitis [2]. A large investigation of 1028 cases highlighted that osteoarticular involvement was the most frequent in human brucellosis and was associated with the highest relapse rate [3]. Likewise, a study presenting the clinical features of brucellosis in Lebanon discovered that osteoarticular involvement of the spine was the most commonly encountered complication, as spondylodiscitis occurred in 44% of 88 analyzed cases [4]. However, noncontiguous multifocal spinal involvement is an exceptional presentation of brucellosis; additionally, an associated paravertebral abscess is extremely rare. To the best of our knowledge, no case of spondylodiscitis with abscess attributable to *Brucella* has been reported in Lebanon. In this paper, we describe a case of noncontiguous multifocal *Brucella* spondylodiscitis, involving the T12–L1 and L3–L4 segments, with the formation of a paravertebral abscess. The patient has benefited from conservative management, responding well to antibiotic treatment.

## Case presentation

A 67-year-old Lebanese woman presented to Notre Dame des Secours University Hospital (Jbeil, Lebanon) on 8 July 2021, with a 3-week history of acute severe lumbar back pain, radiating to the lower extremities and associated with impaired mobility and lower extremity weakness. It is noteworthy that the patient reported having chronic lumbar pain for the past 3 months, yet the last few weeks were marked by hyperalgesia without a history of trauma. She complained of night sweating but had no fever, chills, cough, or dyspnea. However, the patient recalled a 1-week history of fever 1 month ago, after taking the second dose of the Pfizer–BioNTech coronavirus disease 2019 (COVID-19) vaccine. She was admitted to the hospital on the same day, and was referred to the Department of Infectious Diseases for adequate investigation and management. The patient was known to have hypertension, diabetes, dyslipidemia, and hypothyroidism—all treated with medications. She also had a history of lumbar L4–L5 disc herniation, for which arthrodesis was performed 4 years ago. She reported a history of unpasteurized dairy product consumption. Owing to the fact that Lebanon is an endemic area for brucellosis [4], the presence of chronic symptoms with a recent exacerbation shed light on the plausibility of *Brucella* infection with spinal involvement. On physical examination, her temperature was 36.8 °C, respiratory rate 15 breaths per minute, blood pressure 150/80 mmHg, and pulse 105 beats per minute. We noted a painful percussion of the lumbar spine at L3/L4 as well as painful passive mobilization and lumbar paravertebral tenderness. No lymphadenopathy, hepatomegaly, or splenomegaly could be observed. On neurological examination, the range of motion was not decreased for lower extremities; neither sensory deficits nor sphincter abnormalities were present.

**Fig. 1:**
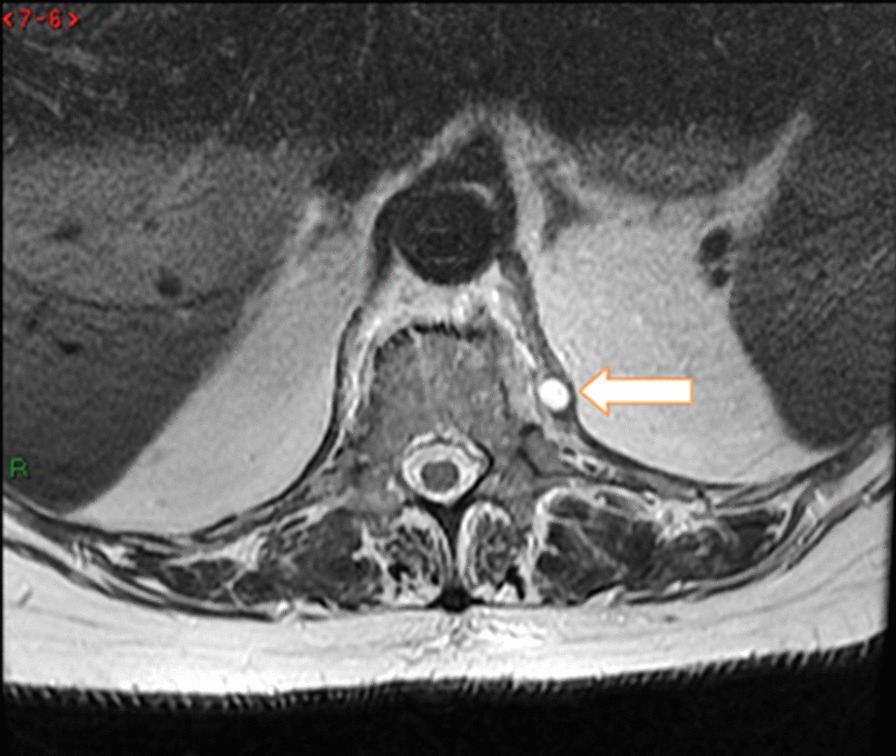
Axial T1 fat-saturation (FS) injected magnetic resonance imaging showing a left paravertebral collection adjacent to T12 (arrow)

**Fig. 2:**
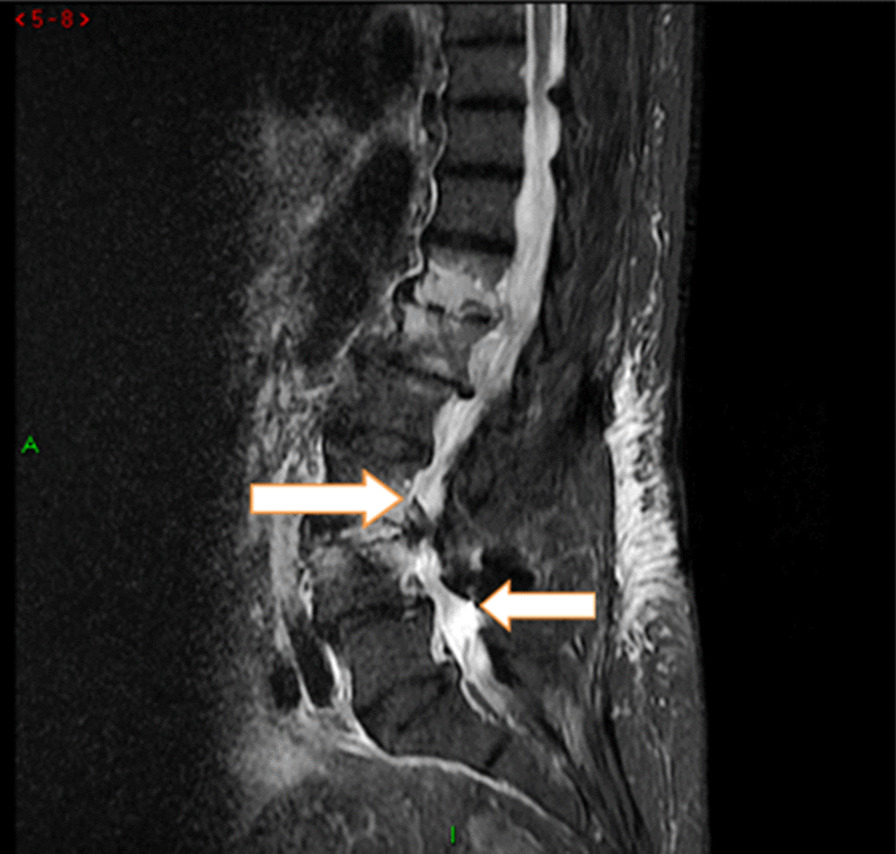
Sagittal stir magnetic resonance imaging showing an abnormal T12–L1 and L3–L4 vertebral body signal with epidural thickness (arrows)

**Fig. 3:**
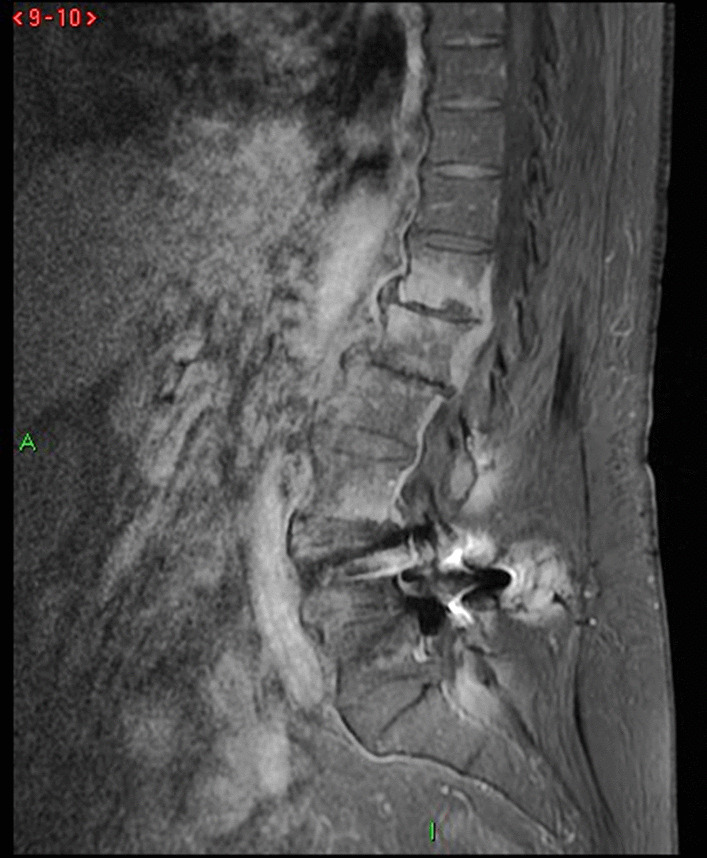
Sagittal injected magnetic resonance imaging showing high epidural and prevertebral soft tissue enhancement

The performed blood tests were indicative of inflammation: hemoglobin 9.4 g/dl, erythrocyte sedimentation rate (ESR) 100 mm per hour, and C-reactive protein (CRP) 70 mg/l. White blood cell count was 6410/μl, and leukocyte formula showed 56% neutrophils, 33% lymphocytes, and 7% monocytes. Blood chemistry was within the normal values. Bacterial serology (BrucellaCapt test) indicated that anti-*Brucella* antibodies were positive at a titer of 1/5120 (reference range 1/180). Magnetic resonance imaging (MRI) of the lumbar spine, performed on 9 July 2021, revealed a multifocal spondylodiscitis involvement of the T12–L1 and L3–L4 discs and their respective adjacent vertebral bodies, with epidural thickening, significant foraminal narrowing at L3–L4 level, and the formation of a paravertebral abscess of 9 mm next to T12. No nerve root compression lesions of the spine were identified. The MRI also showed a grade I anterolisthesis (spondylolisthesis) at L4–L5 (Figs. 1, 2, and 3). Magnetic resonance imaging of the lumbar spine used a high field of 1.5 T.

**Fig. 4:**
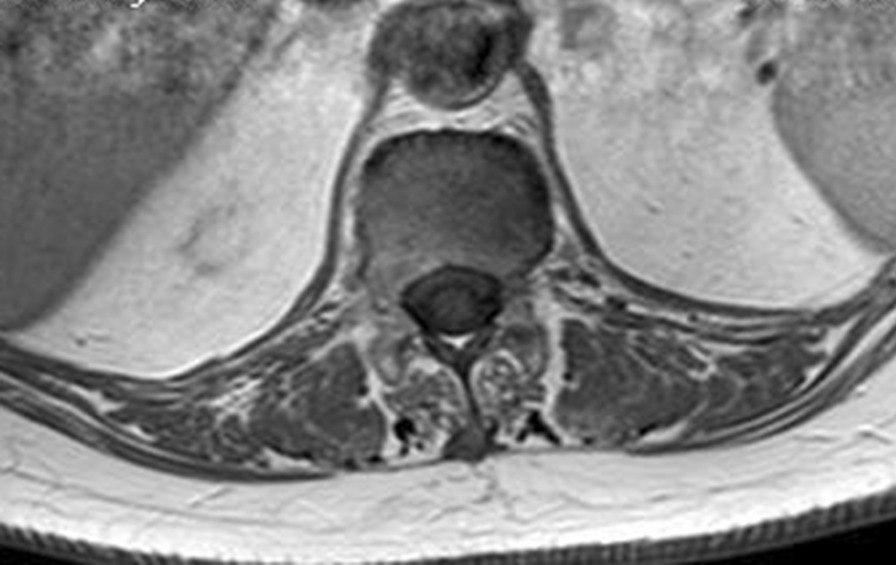
Axial T1 injected magnetic resonance imaging showing a complete resolution of the paravertebral abscess that was originally adjacent to T12

**Fig. 5:**
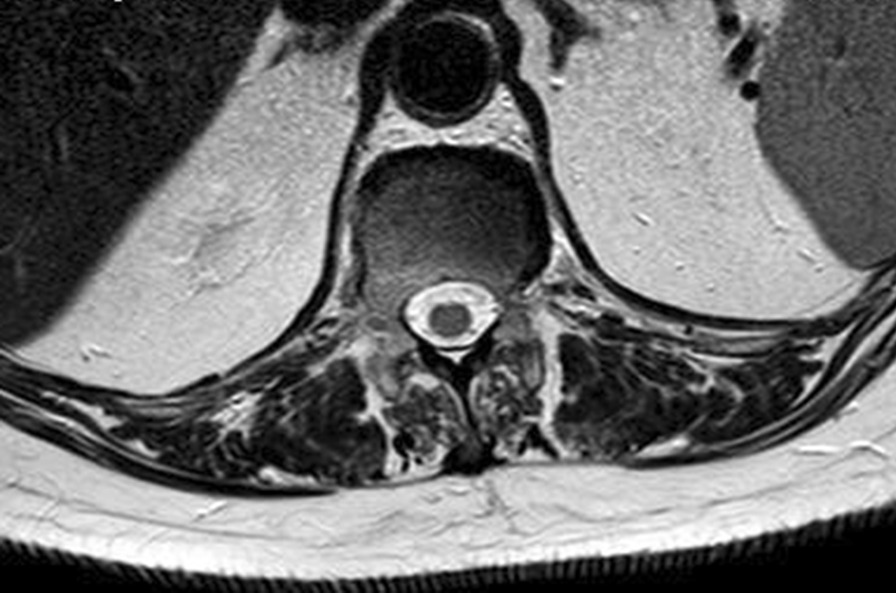
Axial T2 magnetic resonance imaging showing a complete resolution of the paravertebral abscess that was originally adjacent to T12

**Fig. 6:**
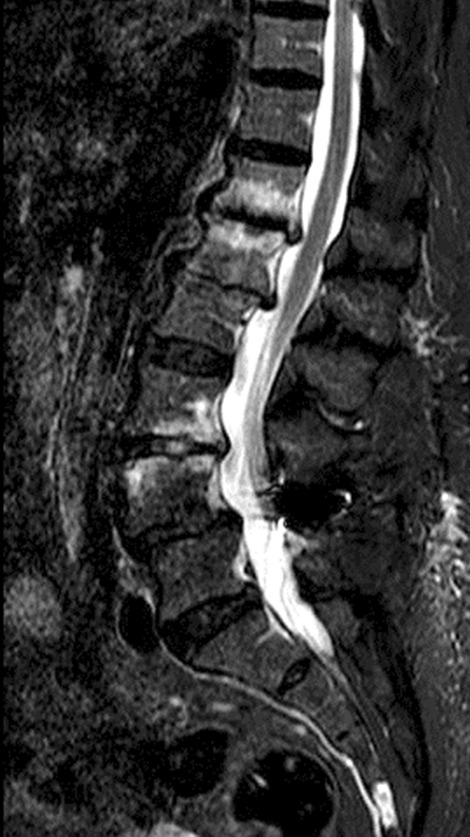
Sagittal stir magnetic resonance imaging

**Fig. 7:**
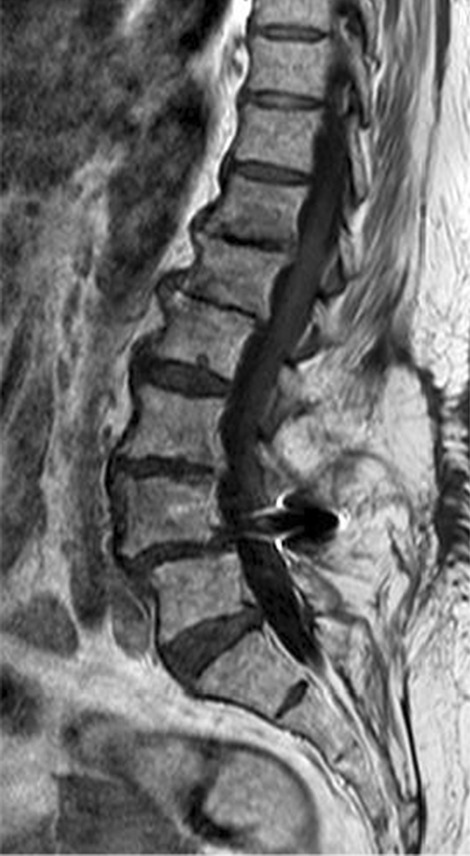
Sagittal T1 injected magnetic resonance imaging

The diagnosis of *Brucella *spondylodiscitis was set. The patient was treated with an inpatient regimen of doxycycline 100 mg by oral route every 12 hours, intravenous ciprofloxacin 400 mg every 12 hours, and intravenous gentamicin 480 mg per day for 2 weeks, which was followed by an outpatient oral antibiotic regimen with doxycycline 100 mg every 12 hours, rifampin 900 mg per day, and ciprofloxacin 500 mg every 12 hours to complete a total treatment duration of 3 months. The patient subsequently experienced a complete resolution of her symptoms and became completely healthy. As part of her follow-up, blood tests were done, indicating normal C-reactive protein and erythrocyte sedimentation rate. An MRI was also performed at the end of the treatment and showed a complete resolution of the paravertebral abscess (Figs. 4, 5, 6, and 7). Magnetic resonance imaging of the lumbar spine using a high field of 1.5 T was performed at the end of the treatment.

## Discussion

The high mutability in clinical forms of brucellosis has become evident in the literature, highlighting the challenge of establishing the diagnosis. A study gathering clinical, laboratory, and therapeutic features of brucellosis in a large series of 480 patients discovered that the most frequent symptoms were malaise, sweating, arthralgia, and back pain, whereas the most common signs were fever, hepatomegaly, splenomegaly, and osteoarticular and nervous system involvement—mimicking a variety of diseases [5]. Elevated erythrocyte sedimentation rate, lymphocytosis, anemia, and—to a lesser extent—thrombocytopenia, leukopenia, and leukocytosis were all associated findings. However, cultures were positive in only 45% of the patients. Therefore, several studies and reports have prompted the consideration of vertebral involvement in brucellosis as a differential diagnosis among subjects presenting with spinal pain in endemic regions, especially when aged above 50 years [5]. In reality, the differential diagnosis of chronic low back pain is vast and could include malignancy, inflammatory diseases, degenerative disc disease or disc herniation, radiculopathy, spinal stenosis, osteoporosis, or sacroiliac joint dysfunction, as well as other infections (for example, tuberculosis) [6]. In relation to our present case, the patient had no symptoms other than night sweats and low back pain; the paucity of symptoms related to brucellosis could have hampered the diagnosis if the suspicion of the disease had not been raised. Regarding imaging, magnetic resonance imaging (MRI) is considered the diagnostic “gold standard” in spondylodiscitis induced by brucellosis [7]; MRI findings can be observed within a month following the first symptoms [8].

Spinal brucellosis may present in two forms: focal or diffuse. The focal form is characterized by the confinement of the infection to the anterior aspect of the superior end plate; in this case, bony destruction extends to a small area. In the diffuse form, the organism affects the whole concerned vertebra and spreads to the adjacent one [9]. Literature has shown that tuberculous spondylodiscitis involving multiple vertebral bodies is a familiar occurrence [10], whereas in brucellosis, unifocal involvement of vertebral bodies/segments is the most frequently seen presentation. Multifocal and multilevel involvement is an exceptional presentation that may be seen in a only minority of cases of *Brucella* spondylitis/spondylodiscitis [8, 11], mainly involving contiguous vertebrae. Nevertheless, rare cases of noncontiguous multifocal spinal involvement of *Brucella* spondylodiscitis have been documented [12–17]. All the patients were males over 60 years of age who reported a history of unpasteurized milk/dairy product ingestion or a direct exposure to dairy animals, and most frequently presented with night sweating, fever, and weight loss [17]. Recently, in 2020, three cases of noncontiguous multifocal *Brucella* spondylitis were reported in China, two 64-year-old and 51-year-old men and a 59-year-old woman, but no associated discitis or abscess could be detected [18].

In fact, when affecting the vertebrae, the infection may spread to surrounding tissues, namely psoas muscles and paravertebral and epidural spaces. Therefore, paravertebral and/or epidural abscesses occur in spondylodiscitis and may mimic disk herniation, but they are also more frequently seen in tuberculous infections compared with brucellosis [11]. Vertebral localization of brucellosis has been accompanied by fever, pain, limited patient ambulation, spinal/peridural abscesses, and spinal cord compression (rarely leading to paraplegia) [19, 20]. Occurrences of paravertebral abscesses complicating *Brucella* spondylitis have also been reported [16, 20–22]. Nevertheless, *Brucella* spondylodiscitis, which is the co-occurrence of discitis and spondylitis, associated with the formation of a paravertebral abscess is rarely observed [23]; a paucity of cases were first described in Turkey and Italy [24–26]. In addition, a unique case was reported in Belgium, where two paraspinal abscesses (from C2–C3 and T1–T2; with epidural involvement) were found in a 79-year-old woman with noncontiguous multilevel spondylodiscitis due to brucellosis [12]. She had symptoms of dorsal medullar compression (manifested as left hemihypoesthesia, left-hand paresis, and left Babinski sign) as well as left scapular pain, as a result of her cervical epidural abscess. The patient was successfully treated with the combination of doxycycline + rifampicin and prednisone, as she remarkably recovered from her pain and part of her motor and neurological deficits [12]. On this basis, *Brucella* spondylodiscitis should be a considered as a perilous complication, as it might be associated with abscesses and neurological complications.

Thereafter, a prospective study was conducted in Turkey that compared patients with and without abscesses in *Brucella* spondylodiscitis [27]. Thirty-one patients had spondylodiscitis and were included in the analysis (23% of the total 135 analyzed cases of brucellosis), and in 19 (61.3%) of them abscesses were identified on magnetic resonance imaging. It is noteworthy that low hemoglobin levels were found in those patients [27], in line with the present case (hemoglobin 9.4 g/dl). Patients were treated medically by a combination of aminoglycoside (that is, streptomycin; the first 3 weeks only), tetracycline ( that is, doxycycline), and rifampin. Significantly higher clinical and radiological improvements were noted among patients without abscesses, compared with their counterparts, after 12 weeks of medical therapy. In addition, longer courses of treatment were required for patients with an abscess; nonetheless, surgical intervention was implemented in no more than two of them, who were both females presenting with neurological deficit and high-grade fever [27].

Likewise, another retrospective multicentric Turkish study endeavored to evaluate the efficacy and optimal duration of treatment in spinal brucellosis, analyzing 293 cases with and without complications [28]. This study found that a paravertebral abscess complicated 13% of cases of *Brucella* spondylodiscitis, followed by epidural (10.2%), prevertebral (4.4%), and psoas abscesses (3.4%), whereas radiculitis was found in 2.7% of the patients. The group with complications exhibited more pronounced clinical presentations in terms of fever and weight loss, and had more perturbed blood tests (for example, higher erythrocyte sedimentation rates, C-reactive protein levels, leukocyte and platelet counts, and lower hemoglobin levels). Thoracic spine involvement was more common in complicated cases [28]. Regarding the evaluation of the treatment effectiveness, no significant disparities were detected between the five applied combination regimens, which were mainly doxycycline and rifampin with/without an aminoglycoside as follows: 1-doxycycline + rifampicin + streptomycin, 2-doxycycline + rifampicin + gentamicin, 3-doxycycline + rifampicin, 4-doxycycline + streptomycin, and 5-doxycycline + rifampicin + ciprofloxacin. The treatment outcomes were identical for the group with complications and that with no complications, although the complicated cases were treated for longer periods of time [28].

Researchers had previously demonstrated that treatments combining doxycycline and streptomycin (with the optional addition of rifampin) were the most effective in cases of osteoarticular involvement due to brucellosis [3]. Surgery of decompression might be necessary in instances of extradural abscess engendering neurological deficits [29], but it is the last option in brucellosis of the spine, as the main treatment is conservative and consists of antibiotics and eventual immobilization [30].

Indeed, there is further evidence that not every patient presenting with spinal brucellosis complicated by an abscess will need surgical treatment. To exemplify, a retrospective study conducted over a 25-year period in Portugal was able to identify 19 cases with abscesses (29.6%) among 54 patients with *Brucella* spondylodiscitis. Eight of the abscesses were paravertebral, yet a single patient with paravertebral abscess underwent surgery for drainage as a result of neurological impairment [31]. Another study concluded that, in the absence of surgical interventions, antibiotic treatment should be implemented until paravertebral or epidural abscesses completely disappear [32].

Paravertebral and spinal epidural abscesses (and occasionally, granulation tissue formation in the epidural space) are seen particularly in cervical spinal brucellosis, compared with other localizations [33]. Although they are very rare complications in cervical localizations, they may have serious consequences, including permanent neurological sequelae and life-threatening vascular/neurological complications [9, 34–37]. Cases of spinal epidural abscesses in the lumbar/lumbosacral regions leading to cauda equina syndrome have also been reported in China and Iran [38, 39]. Moreover, an exceptional case of lumbar *Brucella* spondylodiscitis—with concomitance of an epidural abscess and a large paraspinal abscess extending from L4 to the sacrum—was recently described in Iran: a 21-year-old woman complaining of a 1-year history of severe lumbar back pain but who had no neurological deficit on examination; she was treated with percutaneous drainage, a minimally invasive technique, in addition to antimicrobial therapy [40].

The prognosis of *Brucella* spondylodiscitis appears to be good when the condition is managed adequately [41], but early detection of complicated cases is crucial to circumventing the occurrence of serious complications and sequelae [28]. Nonetheless, as previously discussed, the principal challenge to an efficient management is establishing the diagnosis of *Brucella* in the first place. It is suggested that primary prevention remains vital in endemic regions [41].

## Conclusion

Noncontiguous multifocal *Brucella* spondylodiscitis with paravertebral abscess is an extremely rare presentation. According to our sources, this disease is seen as life-threatening and should be treated aggressively. In relation to our case, it may be effectively managed by antibiotic therapy, without surgery or drainage, in the absence of neurological complications. In endemic countries, a strong suspicion of spinal involvement of brucellosis should be elicited in front of back pain presentations—even in the absence of fever and other related symptoms.

## Data Availability

The authors do not have the right to share any data information as per their institutions’ policies.

## Abbreviations

ESR: Erythrocyte sedimentation rate
CRP: C-reactive protein
MRI: Magnetic resonance imaging
